# Integrated Analysis of Hi-C and RNA-Seq Reveals the Molecular Mechanism of Autopolyploid Growth Advantages in Pak Choi (*Brassica rapa* ssp. *chinensis*)

**DOI:** 10.3389/fpls.2022.905202

**Published:** 2022-06-24

**Authors:** Huiyuan Wu, Xiaoming Song, Shanwu Lyu, Yiming Ren, Tongkun Liu, Xilin Hou, Ying Li, Changwei Zhang

**Affiliations:** ^1^State Key Laboratory of Crop Genetics and Germplasm Enhancement, Key Laboratory of Biology and Germplasm Enhancement of Horticultural Crops in East China, Ministry of Agriculture, Nanjing Agricultural University, Nanjing, China; ^2^Center for Genomics and Bio-Computing, School of Life Sciences, North China University of Science and Technology, Tangshan, China

**Keywords:** RNA-seq, Hi-C, A/B compartment, TADs, autopolyploid, growth advantage, pak choi (*Brassica rapa* ssp. *chinensis*)

## Abstract

Polyploids generated by the replication of a single genome (autopolyploid) or synthesis of two or more distinct genomes (allopolyploid) usually show significant advantages over their diploid progenitors in biological characteristics, including growth and development, nutrient accumulation, and plant resistance. Whereas, the impacts of genomic replication on transcription regulation and chromatin structure in pak choi have not been explored fully. In this study, we observed the transcriptional and genomic structural alterations between diploid *B. rapa* (AA) and artificial autotetraploid *B. rapa* (AAAA) using RNA-seq and Hi-C. RNA-seq revealed 1,786 differentially expressed genes (DEGs) between the diploids and autotetraploids, including 717 down-regulated and 1,069 up-regulated genes in autotetraploids. Of all the 1,786 DEGs, 23 DEGs (10 down-regulated DEGs in autotetraploids) were involved in Compartment A-B shifts, while 28 DEGs (20 up-regulated DEGs in autotetraploids) participated in Compartment B-A shifts. Moreover, there were 15 DEGs in activated topologically associating domains (TADs) (9 up-regulated DEGs in diploids) and 80 DEGs in repressed TADs (49 down-regulated DEGs in diploids). Subsequently, eight DEGs with genomic structural variants were selected as potential candidate genes, including four DEGs involved in photosynthesis (*BraA01003143*, *BraA09002798*, *BraA04002224*, and *BraA08000594*), three DEGs related to chloroplast (*BraA05002974*, *BraA05001662*, and *BraA04001148*), and one DEG associated with disease resistance (*BraA09004451*), which all showed high expression in autotetraploids. Overall, our results demonstrated that integrative RNA-seq and Hi-C analysis can identify related genes to phenotypic traits and also provided new insights into the molecular mechanism of the growth advantage of polyploids.

## Introduction

Polyploids have three or more intact sets of chromosomes per nucleus, which are produced by intraspecific genome doubling (autopolyploid) or merging genomes of distinct species by hybridization (allopolyploid) (te Beest et al., 2012). Polyploidization takes an essential part in the process of biological evolution, about 70% of angiosperms have undergone one or more polyploidization in their evolutionary history (Fawcett and Van De Peer, 2010). Meanwhile, polyploids have a variety of superior advantages over their diploid ancestors, which exhibit in phenotypic and physiological characteristics, including stronger tolerance, higher content of active compounds, increased organic synthesis rate, and enlarged organs (Fawcett et al., 2009). For instance, the fruit sizes of the autotetraploid kiwifruit and grape are significantly increased (Wu et al., 2012), and the fruit quality is elevated in autotetraploid watermelons (Jaskani et al., 2005). With the chromosome doubling, the number of alleles per locus on the chromosome doubles, resulting in changes in the quality and quantity of gene expression (Hegarty et al., 2008). Research has shown that polyploidization may affect the expression of various functional genes, but the frequency of genes involved in cell defense and senescence, plant hormone regulation, and metabolism is relatively high, indicating that the effect of polyploidization on gene expression is not random (Wang et al., 2006).

Chromosomes in eukaryotes composed of histones together with highly compressed and folded linear DNA are not randomly packaged in the nucleus (Tanay and Cavalli, 2013). The importance of chromatin arrangement in transcription regulation has been gradually revealed (Vergara and Gutierrez, 2017). As currently the most comprehensive and high-throughput technology, Hi-C (Lieberman-Aiden et al., 2009) can divide interaction frequencies between almost any pair of genomic sites, which has been used to investigate genome architecture in three dimensions in various plant species, including *Arabidopsis* (Feng et al., 2014), maize (Sun et al., 2020), rice (Liu et al., 2017; Dong et al., 2018), cotton (Wang et al., 2018), and tomato (Wang et al., 2020). The majority of eukaryote genomes are structured into hierarchical layers, including chromosome A/B compartments at the megabase scale, topologically associating domains (TADs) at the sub-megabase scale (Rao et al., 2014; Wang et al., 2016). Each layer is not static, but all exhibit remarkable flexibility and inherent property to dynamically reorganize. Compartment A/B can distinguish the expression levels of genes in cells under different conditions. In general, the A compartment contains highly transcribed genes with active histone modifications. Analogously, the B compartment contains inactive genes with histone modifications indicating transcriptional repression (Lieberman-Aiden et al., 2009). The conversion of Compartment A to B may cause the gene to change from active expression to gene silencing, and vice versa (Dixon et al., 2015). For example, Compartment A/B shifts between diploid and autotetraploid *Arabidopsis* resulted in a transcriptional change (Zhang et al., 2019). The genome multiplication of watermelon resulted in 108 A-to-B and 626 B-to-A compartment shifts that contained stress-related and growth-related genes (Garcia-Lozano et al., 2021).

The TAD (topologically associating domain) is the basic organization of the genome in spatial structure and shows a significant similarity in different species (Le Dily et al., 2014). TADs are generally referred to as “squares” of interaction with “specific biological functions,” where the contact between two nearby regions is far less often than interaction within the region (Ulianov et al., 2016). TAD boundary is enriched with insulator-binding protein CTCF, a housekeeping gene, tRNA, and adhesion protein, which contributes to the structural and functional stability of TAD. The change of this boundary will cause a disorder in gene regulation (Nora et al., 2012). Wang et al. (2021) discovered that polyploidization in soybean could cause TAD architecture changes and that subsequent diploidization altered TADs around chromosome-rearrangement sites. Additionally, altered TADs in autotetraploid cotton affected the transcriptional activity of a large number of genes compared to diploids (Wang et al., 2018).

As a member of the *Brassica* family, non-heading Chinese cabbage (*Brassica rapa* ssp. *chinensis*), also known as pak choi, is one of the most popular vegetable crops and is commonly grown in East Asia with high nutritional and commercial values (Song et al., 2020). A previous study has revealed that autotetraploid pak choi possess greater organs than their diploid progenitor (Zhang et al., 2020). Notwithstanding numerous studies that have evaluated the impacts of transcriptomic changes resulting from genome duplication in other species (Adams et al., 2003; Roulin et al., 2013; Hovav et al., 2015), little is known in terms of pak choi. To shed light on this problem, we conducted an RNA-seq of diploid and autotetraploid pak choi to obtain whole transcriptome expression profiles and Hi-C sequencing was used to characterize the three-dimensional genomic structure. Taken together, this study provides novel insights into not only 3D genome architecture but also the identification of candidate genes in plants, as well as the comprehension of how autotetraploidy enhances plant development performance.

## Materials and Methods

### Plant Materials, Growth Conditions, and Sample Collection

The diploid non-heading Chinese cabbage of *B. rapa* (AA) accessions, “Wuyueman” (WYM 2, an elite cultivar in Yangzi River of China), and its colchicine-induced stable autotetraploid of *B. rapa* (AAAA) accessions “Wuyueman” (WYM 4), were cultivated in the greenhouse of Nanjing Agricultural University in plastic pots with peat fertilizer and soil at a ratio of 1:1. A fully expanding young leaf (3rd upper leaf of a plant) was respectively collected from each individual seedling of the two species with three biological replicates and immediately immersed in liquid nitrogen, then stored at −80°C for following experiments.

### RNA-Seq Libraries Preparation and Sequencing

The total RNA of six samples was extracted from the frozen leaves of WYM 2 and WYM 4 using an RNAprep Pure Plant kit (Tiangen, Beijing, China) in accordance with the manufacturer’s procedure. RNA quality and purity were verified using a NanoDrop 2000 spectrophotometer (Thermo Fisher Scientific, United States), and RNA integrity was assessed by 1% agarose gel electrophoresis. The creation of cDNA libraries and transcriptome sequencing were conducted by Novogene Biotechnology Co., Ltd on the Illumina Novaseq platform and 150 bp paired-end reads were obtained. Following quality assurance, the screened clean reads were then aligned to the reference genome (*B. rapa* genome), which was obtained from the BRAD database^1^ using the HISAT2 (version 2.1.0) (Kim et al., 2015). Cufflinks was used to assess the amount of gene expression. FPKM (fragments per kilobase of exon per million fragments mapped) was used to quantify gene expression (Trapnell et al., 2012).

### Functional Enrichment Analysis of Differentially Expressed Genes

DESeq2 R software was used to do differential gene expression analysis. The thresholds for identifying DEGs were established at the false discovery rate (FDR) < 0.01 and | log_2_ FC| > 1 and adjusted *P*-value < 0.05. The Gene Ontology (GO) and Kyoto Encyclopedia of Genes and Genomes (KEGG) databases were searched for annotation to determine probable biological functions and pathways for the DEGs. The GO enrichment analysis of DEGs was carried out in R using the GOseq package (Young et al., 2010). The cluster Profiler R program (Yu et al., 2012) was employed to investigate the enrichment of DEGs in KEGG pathways.

### qRT-PCR Analysis

The RNA-seq data was confirmed by qRT-PCR analysis of fifteen DEGs selected randomly. cDNA was synthesized from 1 μg of total RNA by *Evo M-MLV* RT Mix Kit with gDNA Clean for qRT-PCR [AG11728, Accurate Biotechnology (Hunan) Co., Ltd], then analyzed by qRT-PCR using Hieff^®^ qPCR SYBR^®^ Green Master Mix (High Rox Plus) (Cat No. 11203ES08; Yeasen, Shanghai, China) on StepOnePlus system (Applied Biosystems, United States). *BrActin* (*Bra028615*) was used as a quantitative reference (Dheda et al., 2004). Primer 6.0 software (Premier, Ottawa, Canada) was used to design the corresponding primers, which are listed in Supplementary Table 3. Each 20 μL PCR reaction comprised 10 μL of 2× SYBR Green Master Mix, 0.4 μL of 10 μM primers, 2 μL of cDNA template, and 7.2 μL of ddH_2_O. qRT-PCR was as follows: 95°C 5 min, 95°C 10 s, 60°C 30 s, 40 cycles. Each sample had three biological and three technical duplicates. The 2^–ΔΔCt^ approach was applied to quantify relative gene expression levels (Schmittgen and Livak, 2008).

### Hi-C Library Construction and Sequencing

Creating Hi-C libraries and completing the Illumina sequencing were conducted by Novogene Company in China. The library construction was prepared according to the standard protocol described previously with certain modifications (Van Berkum et al., 2010). After the completion of the sample of crosslinking and cell lysis, the samples were digested with DPN II. Then marked the end with biotinylated nucleotides and added the connection. Protease K and SDS were added to reversely cross-linked. Subsequently, ligated DNA was purified and fragmented into 300 bp size on average. Finally, Illumina HiSeq was used for sequencing after the constructed library was qualified by library quality control.

### Processing Hi-C Data for Construction of Hi-C Interaction Maps

The output data of Hi-C sequencing was first quality controlled by filtering out the paired reads including sequences with adapter contamination, unidentified nucleotides “N” ratio >10%, and more than 50% base with Q <5. Next, the qualified reads of Hi-C data were processed by following the HiCUP pipeline (v0.57) (Wingett et al., 2015). Simply, after the qualified reads were truncated with the DpnII restriction enzyme, the resulting trimmed reads were then, respectively, aligned against the *B. rapa* genome (v2.5) (Cai et al., 2017) by employing the Bowtie 2 (v2.2.3) program (Langmead and Salzberg, 2012) with default parameters and obtain Hi-C contact matrices. The MDS algorithm of PASTIS software (Szałaj et al., 2016) was employed to imitate the 3D position of chromatin.

### A/B Compartment and Topologically Associating Domains Analysis

Principal component analysis (PCA) of interaction maps with a resolution of 100 Kb was used to identify chromosomal compartments (Imakaev et al., 2012). The “runHiCpca.pl” script of the HOMER Package was used to perform PCA analysis (Niskanen et al., 2018). In order to examine distance normalized contact normalization, we began by determining the optimal background model. Then we constructed contact matrices on every chromosome as well as calculated the correlation coefficient between each region and other regions. Lastly, based on the correlation matrix, we calculated the first principal component whose eigenvalues were used to divide the compartments into two types. Positive and negative eigenvalues indicate compartments A and B, respectively.

TadLib (hitad 0.1.1-r1) software was applied to estimate the TAD topology at a 40 Kb resolution with default parameters. RNA-seq was performed on the genes in TADs, and we classified TADs into three categories based on the proportion of genes with positive and negative FC in each TAD of AA vs. AAAA: activated TADs, repressed TADs, and other TADs.

## Results

### Overview of RNA-Seq Data of Diploid (AA) and Artificial Autotetraploid (AAAA) *B. rapa*

**FIGURE 1:**
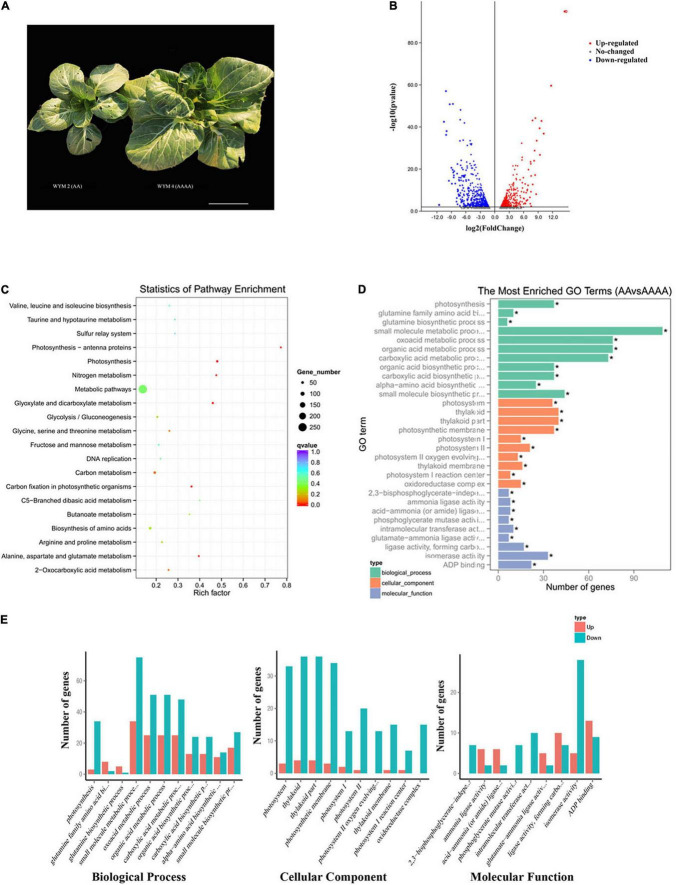
RNA-seq analysis of diploid (AA) and autotetraploid (AAAA) pak choi. **(A)** Phenotypic differences. Scale bar = 10 cm. **(B)** Volcano map of DEGs. Red points denote up-regulated genes; blue points denote down-regulated genes; gray points denote non-differentiated genes. **(C)** The top 20 enriched KEGG pathways of DEGs. The pathway label is on the vertical axis, the Rich factor is on the horizontal axis, the size of the dots represents the number of DEGs in the pathway, and the color of the dots represents the distinct *Q*-value levels. **(D)** The most enriched GO terms with all DEGs. The ordinate is the enriched GO term, the abscissa is the number of DEGs in the term, and GO terms with “*” are significantly enriched. **(E)** The most enriched GO terms with up-regulated DEGs and down-regulated DEGs.

As shown in Figure 1A, autotetraploids had a greater overall plant size and single leaf area than diploids. A previous study has revealed that the whole plant height, weight, stomatal size, flower, pod, and seed size of autotetraploid pak choi were all more significant than diploids. Furthermore, autotetraploid pak choi exhibited enhanced specific leaf weight (SLW), indicating a faster rate of photosynthesis (Zhang et al., 2020). In view of the results of the previous study, we performed RNA-seq on the diploid (AA) and artificial autotetraploid (AAAA) pak choi. 40.96 Gb raw reads of six cDNA libraries in total were generated for RNA-seq. After filtering adapter and low-quality reads, for each library, the proportion of clean reads was greater than 98% and GC ratios ranged from 46.14 to 47.27%. The Q30 was ≥93.03% and Q20 was ≥97.56%, which informs the sequencing quality was quite high (Supplementary Table 1). Subsequently, the clean reads were aligned against the *B. rapa* genome, with alignment efficiency ranging from 85.58 to 87.34%. The unique map ratio was between 78.91 and 80% (Supplementary Table 2). The correlation coefficient between replicates within each AA or AAAA was all above 92%, showing high reproducibility between replicates (Supplementary Figure 1). Consequently, 1786 significant changed genes were identified, which included 717 up-regulated and 1,069 down-regulated genes in diploids (Supplementary Table 4). In addition, the proportion of low expressed genes was higher in autotetraploids (Supplementary Figure 2). Volcano plots (Figure 1B) were generated to summarize the significant DEGs.

### Gene Ontology and Kyoto Encyclopedia of Genes and Genomes Analysis of the Differentially Expressed Genes

To functionally annotate the DEGs, we used the GO and KEGG databases to align all of the DEGs with a *Q* value <0.05. There were 39 statistically significant enrichment GO terms among the DEGs (Supplementary Table 5). Figure 1D showed the top 30 enriched GO terms. In the biological process group, DEGs were significantly enriched in terms related to metabolic pathway, among which the “small molecule metabolic process” (GO: 0044281, 109 DEGs) was enriched with the most DEGs, followed by “oxoacid metabolic process” (GO: 0043436, 76 DEGs) and “organic acid metabolic process” (GO: 0006082, 76 DEGs). Within the cellular component, terms associated with thylakoid, such as “thylakoid” (GO: 0009579, 40 DEGs) and “thylakoid par” (GO: 0044436, 40 DEGs) were enriched. In terms of the molecular function category, “isomerase activity” (GO: 0016853, 30 DEGs) was prominently represented. These findings point to a high proportion of transcriptome changes following polyploidization, besides, activated genes are more than repressed (Figure 1E).

According to KEGG enrichment analysis, there were 1,303 DEGs mapped to 110 KEGG pathways in total (Supplementary Table 6). Figure 1C shows the top 20 enriched pathways assigned to our DEGs. Most of the DEGs participated in “Metabolic pathways” (258 DEGs, ath01100) and “Biosynthesis of secondary metabolites” (122 DEGs, ath01110), followed by “Carbon metabolism” (47 DEGs, ath01200), “Biosynthesis of amino acids” (44 DEGs, ath01230), “Ribosome” (42 DEGs, ath03010), and “Photosynthesis” (37 DEGs, ath00195).

### Transcriptome Analysis of Differentially Expressed Genes Related to Photosynthesis

Photosynthesis is associated with plant growth and biomass accumulation, which plays an important role in polyploids. Based on the *p*-value, the photosynthetic pathway was the most significantly enriched pathway. Subsequently, we picked 35 DEGs in the photosynthetic pathway, of which only 2 were downregulated in autotetraploids and 16 DEGs involved in photosystem I and 15 DEGs in photosystem II as well as four other DEGs (Supplementary Table 7). The DEGs involved in photosystem I contained *psaN*, *psaG*, *psaE*, *psaF*, *psaH*, and *psaO*. While the DEGs that participated in photosystem II included *PsbQ*, *PsbW*, *PsbP*, *PsbY*, *PsbR*, and *Psb28*.

### Genome-Wide Interaction Matrices of AA and AAAA

We used Hi-C experiments on AA and AAAA to explore the dynamics of 3D genome structure during *B. rapa* polyploidization, then 114 and 206 Gb raw data were generated, respectively (Supplementary Table 8). After mapping against the *B. rapa* (Cai et al., 2017) genome, we got 755 and 1,369 million valid reads for 3D genome construction and the unmappability of the reads was 15.65 and 16.88% for diploids and autotetraploids, respectively (Supplementary Table 9).

**FIGURE 2:**
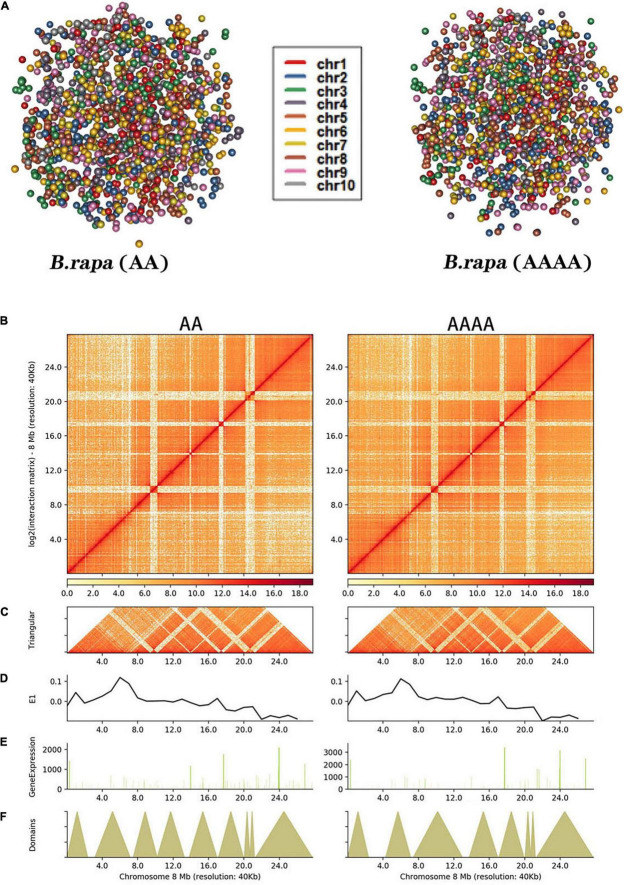
Hi-C analysis of chromatin contacts in diploid and autotetraploid pak choi. **(A)** 3D model of whole chromosomes. Each chromosome is represented by a different hue. **(B)** Intrachromosomal interactions of the chromosome at 40 Kb resolution. **(C)** Each diagonally spread triangle corresponds to a TAD. **(D)** First principal component values show the type of A/B compartment. **(E)** Gene expression level based on RNA-seq. **(F)** Distributions of TADs.

The notion of “chromosome territory,” in which each chromosome has its own private nucleus territory, was supported by genome-wide simulation images, which revealed that chromosomes were positioned in a limited volume (Tanabe et al., 2002; Figure 2A). At a resolution of 1 Mb, both contact maps revealed that intrachromosomal interactions were more pervasive than interchromosomal interactions (Supplementary Figure 3). It also turned out that autotetraploid pak choi had a higher ratio of inter-/intrachromosomal (*trans*/*cis*) contacts than diploid pak choi (Supplementary Figure 5 and Supplementary Table 10). Thus, the greater number of chromosomes in autotetraploids compared to diploids appeared to increase the frequency of interactions. Each chromosome’s space in the nucleus might be altered as a result. For further understanding of the interaction patterns within chromosomes, the interaction matrix of 10 chromosomes in the whole genome interaction matrix of AA and AAAA (Supplementary Figure 4) was constructed at 40 Kb resolution. Take chromosome 8 (Figure 2B) for example, the dark red color of the diagonal indicated the strongest interaction within the same chromosome. With rising horizontal distance, the occurrence of intrachromosomal interactions was reduced. Additionally, more delicate components like TADs could also be found (Figure 2C). Each diagonally distributed triangle corresponded to a topologically associated domain (TAD) (Figure 2F). Meanwhile, there was no significant difference in the distribution of compartments (Figure 2D) and gene expression (Figure 2E) between AA and AAAA.

### Identification of A/B Compartment Shifts

**FIGURE 3:**
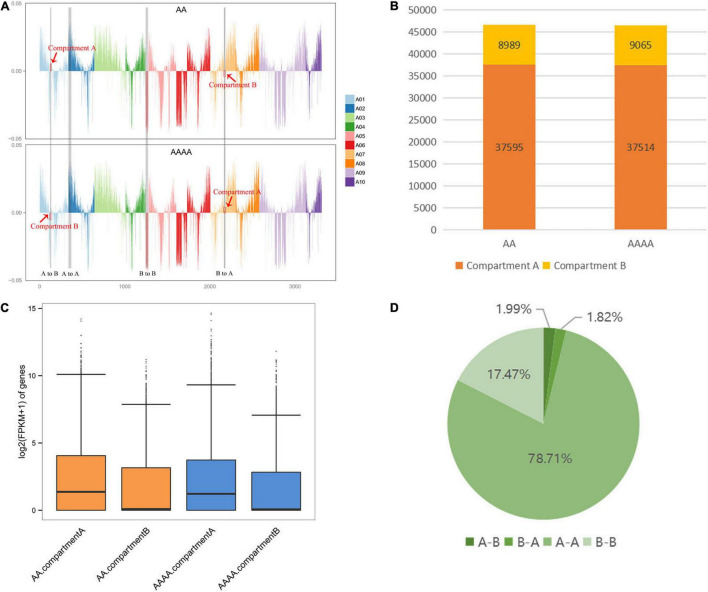
The analysis of the A/B compartments in diploid and autotetraploid pak choi. **(A)** The distributions of the A/B compartment on each chromosome. With the *y*-axis 0 scale line as the reference, above is Compartment A and below is Compartment B. **(B)** The number of genes contained in the A and B compartments. **(C)** Box plots representing the gene expression on the A and B compartments. **(D)** The percentage of the A/B compartment shifts between diploid and autotetraploid pak choi.

In general, Compartments A are euchromatin transcriptional active regions, while Compartments B are heterochromatin transcriptional repressed regions. Gene expression is affected by the conversion between Compartments A and B: Compartments B-to-A shift usually boosts gene expression, and vice versa. Each chromosome’s A/B compartment distribution of AA and AAAA was shown in Figure 3A. In total, 37595 A (80.70%) and 8,989 B (19.30%) compartments were identified in diploids, whereas 37,514 A (80.54%) and 9,065 B (19.46%) compartments were identified in autotetraploids (Figure 3B and Supplementary Table 11). Compared to the B compartments, the A compartments had higher gene intensity, lower GC content (Supplementary Table 8), and a considerable increase in transcription of both two samples (Figure 3C), which was consistent with earlier compartment pattern research in *Arabidopsis* (Grob et al., 2014).

Following genome doubling, there were 1,776 A/B compartment shifts overall, with 926 (1.99%) A-to-B shifts and 850 (1.82%) B-to-A shifts, while a considerable number of compartments remained stable (78.71% conserved A compartments and 17.47% conserved B compartments) (Figure 3D). Combined with transcriptome data, 23 and 28 DEGs were identified in A-to-B shifts and B-to-A shifts, respectively. Screening the 51 DEGs associated with A/B compartment shifts identified 20 (71.43%) DEGs upregulated in the B-to-A compartment and 10 (43.48%) DEGs downregulated in the A-to-B compartment (Supplementary Table 12). The inconsistent relationship suggests that the uncoupling of compartmental variation and gene expression resembles the results in *Drosophila* (Ghavi-Helm et al., 2019).

### Identification of Different Kinds of Topologically Associating Domains

**FIGURE 4:**
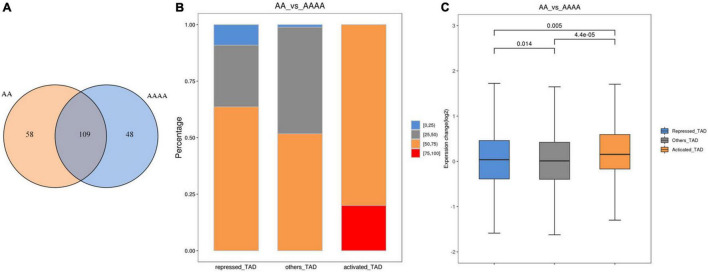
The analysis of TADs in diploid and autotetraploid pak choi. **(A)** Venn diagram of TADs detected in AA and AAAA. **(B)** Proportional stacking maps of genes with different expression levels in different TADs. For each gene contained in the TADs, the change in expression level was calculated for both sets of samples, and the percentage of all genes corresponding to that TAD with Fold Change greater than 1. The percentages were divided into four categories, including 0–25%, 25–50%, 50–75%, and 75–100%. The results for the different categories of TADs were presented in the form of bar charts. **(C)** Gene expression of the three types of TADs.

Topologically associating domains are defined as continuous regions, the frequency of interaction within the region is significantly higher than that of the two adjacent regions (Dixon et al., 2012), and they have been proved to affect transcriptional regulation in various ways (Gibcus and Dekker, 2013; Bonev and Cavalli, 2016). We identified 167 and 157 TADs in AA and AAAA, respectively, of which 109 were conserved (Figure 4A). In this study, we did not detect the difference in the TAD boundary region, so we used previous methods (Le Dily et al., 2014) to predict and classify conserved TADs according to the change in gene expression in TADs after genome doubling. Figure 4B showed that activated TAD had a greater percentage of fold change (AA/AAAA) >1 than repressed TAD. Moreover, the expression change of activated TAD was higher than repressed TAD (Figure 4C). As a result, 10 activated TADs, 11 repressed TADs, and 88 other TADs were identified (Supplementary Table 13). Besides, 15 DEGs were recognized in activated TADs (9 up-regulated DEGs in diploids) and 80 DEGs were identified in repressed TADs (49 down-regulated DEGs in diploids) (Supplementary Table 14).

### Growth Advantage Gene Candidates and Validation of qRT-PCR

**FIGURE 5:**
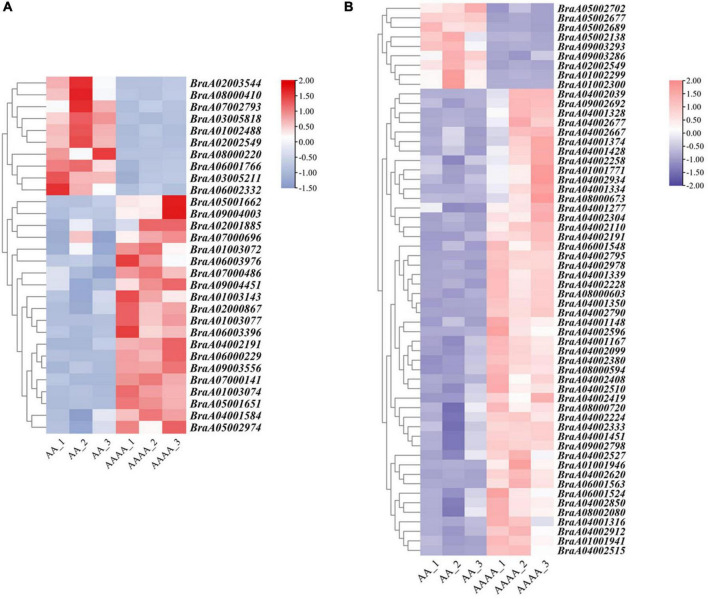
The validation of RNA-seq data. **(A)** qRT-PCR validation of 15 DEGs. *X*-axis: Gene ID; *Y*-axis: log2 (FC), FC (Fold change) represents the ratio of gene expression between AA and AAAA, the logarithm to its base 2 is log2 (FC). **(B)** qRT-PCR and RNA-seq data correlation analysis.

**FIGURE 6:**
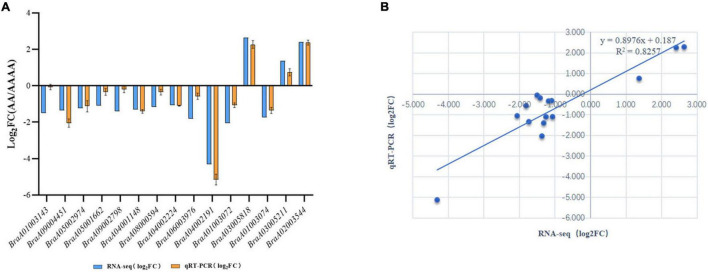
The heat map of gene expression screened by the integrative analysis of RNA-seq and Hi-C. **(A)** The gene expression heatmap of down-regulated DEGs in autotetraploid pak choi involved in A-to-B compartment and up-regulated DEGs involved in B-to-A compartment. **(B)** The gene expression heatmap of down-regulated DEGs in diploid pak choi in repressed TADs and up-regulated DEGs in activated TADs.

The study’s main goal was to uncover the underlying molecular mechanisms of autotetraploid growth advantage by identifying differences between genomic and spatial features. An integrative study of RNA-seq and Hi-C data was used to screen the DEGs that were influenced by 3D reorganization or disarray (Supplementary Table 15). There were 88 DEGs altogether in A/B compartment shifts and activated and repressed TADs (Figure 5). Interestingly, *BraA02002549* and *BraA04002191* were both in A/B compartment and TADs. Furthermore, potential candidate genes were found in eight DEGs with genomic structural variants, including four DEGs involved in photosynthesis, three DEGs related to the chloroplast, and one DEG associated with disease resistance, which may contribute to autotetraploid growth advantage (Table 1). To validate the accuracy of RNA-seq data, qRT-PCR was performed on the 15 DEGs, consisting of seven random DEGs and eight candidate genes. The two methods (qRT-PCR and RNA-sequencing) revealed similar trends in the expression profiles of those 15 DEGs (Figure 6A), and the correlation coefficient of R^2^ = 0.8257 (Figure 6B), indicating the high reliability of the applied RNA-seq analysis.

**TABLE 1:** The candidate genes for growth advantage screened through a comprehensive RNA-seq and Hi-C analysis.

Gene ID	Description	Regulation in AAAA	Type
*BraA01003143*	PSI (Lhca3*1)	Up	CopartmentB-A
*BraA09004451*	homolog of *RPW8*	Up	CopartmentB-A
*BraA05002974*	encodes GDC1 (Grana Deficient Chloroplast 1)	Up	CopartmentB-A
*BraA05001662*	one of four *Arabidopsis* homologs of bacterial ymlg proteins.	Up	CopartmentB-A
*BraA09002798*	light-harvesting complex II chlorophyll a/b binding protein 5	Up	repressed TAD
*BraA04001148*	involved in the escape movement of chloroplasts under high-light environments	Up	repressed TAD
*BraA08000594*	encodes a fructose-1,6-bisphosphatase	Up	repressed TAD
*BraA04002224*	encodes PsbW, a protein similar to PS II reaction center subunit W	Up	repressed TAD

## Discussion

Polyploidization is a rather typical occurrence in plant evolution, and it is also an important way to form new species. Previous studies on polyploids have shown the progeny of polyploid plants typically possess larger organs, higher yields, increased toughness, and other excellent agronomic characteristics relative to their diploid parents (Mu et al., 2012; Tsukaya, 2013). Nevertheless, the underlying molecular mechanisms of the autotetraploid growth advantage are complex and need to be elucidated further. With the rapid development of high-throughput sequencing and plant genome sequencing technologies, it’s more efficient to study transcriptional and regulatory effects across the genome. Many polyploid plants have been studied using RNA-seq, including polyploid *Arabidopsis*, *Brassica napus*, cotton, wheat, and other species (Akhunova et al., 2010; Flagel and Wendel, 2010; Kim and Chen, 2011; Higgins et al., 2012). The genome duplication in pak choi occurs along with changes in gene expression and potential transcriptome modifications. The accurate regulation of gene expression in several layers is dependent on the three-dimensional (3D) structure of chromatin (Bonev and Cavalli, 2016). In this work, we evaluated the 3D genome of diploid and autotetraploid pak choi and combined Hi-C and RNA-seq data.

The GO analysis showed that DEGs were mostly enriched in terms related to photosynthesis and thylakoid. Moreover, the number of activated DEGs was more than inhibited after genome duplication, which indicated that genome duplication promoted the expression of genes for photosynthesis, leading to an autotetraploid growth advantage. To a certain extent, it verified and explained the previous studies (Zhang et al., 2020). According to KEGG significance enrichment analysis, DEGs were mainly active in photosynthesis and photosynthesis antenna proteins, indicating that they played a key role in autotetraploid plant development.

It’s now clear that phenotypic characteristics aren’t determined solely by DNA sequence and the different hierarchies of the 3D genome have the potential to regulate the expression of genes with various levels of regulatory control (Kong and Zhang, 2019). Our results revealed that autotetraploid pak choi had a reorganization of interactions within chromosomes and between different chromosomes, which was studied hierarchically. Furthermore, TADs and compartment condition were proved to be extremely stable between diploid and autotetraploid *B. rapa*. Yet we also found that only 43.48% of DEGs in the A-to-B compartment were repressed in autotetraploid pak choi, illustrating that the majority of this remodeling at the local level is not directly related to changes in gene expression. This finding is in line with recent research indicating that changes in transcription aren’t always accompanied by changes in genomic structure (Ghavi-Helm et al., 2019).

A total of 87 DEGs were revealed by integrative analysis of Hi-C and RNA-seq in this study. It was shown that polyploidy made genes that help the plant grow, develop, and respond to stress more active in pak choi, and the majority of them were concerned with photosynthesis and chloroplasts. As a result, autotetraploid pak choi showed improved carbon fixation and photosynthesis. Finally, we screened eight potential genes that might play a role in the growth advantage from the compartment A/B shifts and the repressed TADs, including four DEGs involved in photosynthesis, three DEGs related to chloroplast, and one DEG associated with disease resistance. Among these DEGs, *BrLHCA3* (*BraA01003143*), *LHCB5* (*BraA09002798*), and *BrPSBW* (*BraA04002224*) are related to the photosystem. The Calvin cycle (CC), photosystem I (PSI), photosystem II (PSII), and the light-harvesting complex (LHC) are the four primary gene networks that mediate photosynthesis, and whole-genome duplications revealed that dosage sensitivity drives photosystem gene family evolution (Coate et al., 2011). Light-harvesting chlorophyll a/b binding protein (LHC) is an important functional protein in the photosynthetic system, which is located on the chloroplast thylakoid membrane (Raghavendra, 2000). In higher plants, *LHC* gene families include *LHCA* and *LHCB* subfamilies, which encode proteins that constitute light collection complexes for light systems I and II (Jansson, 1999). Many studies have shown that *LHCA* and *LHCB* genes play an important role in the adaptation of plants to environmental stress. Fan et al. (2011) found that *LHCA* gene expression was significantly up-regulated in salt-tolerant plants. Cold pressure stimulates the activation of the Lhcb1*3 gene in *Arabidopsis* seedlings cultivated in the dark (Capel et al., 1998). In moss, the absence of *LHCB5* led to disordered thylakoid structure, reduced granular membranes, and increased starch granules (Peng et al., 2019). In *Arabidopsis thaliana*, *LHCB5* knock-out led to decreased energy-transfer efficiency from the LHCII (light-harvesting complex II) to the PSII reaction center (Chen et al., 2018). PsbW is a nuclear-encoded protein expressed in the chloroplast’s thylakoid membrane (Shi and Schröder, 1997). The loss of *PsbW* in *Arabidopsis* destroyed the stability of PSII supramolecular structure, which led to a slight decrease in the chlorophyll fluorescence parameter FV/FM, a significant decline in the phosphorylation of the PSII core protein, and a modification of the plastoquinone (PQ) pool’s redox state in leaves that have acclimated to darkness (García-Cerdán et al., 2011). As mentioned, previous studies comparing polyploids to diploids in pak choi observed higher photosynthetic rates in polyploids (Zhang et al., 2020), which we speculated may be related to the three candidate genes. However, we do not yet clearly know how these candidate genes work in *B. rapa*, and further studies are needed. In addition, the correlative findings require experimental validation, such as genome editing, to confirm potential relationships among genome alterations, the 3D genome, and gene expression regulation in genome duplication.

## Conclusion

It is possible that the rebuilding of 3D genomic architectural maps, as well as the location of variably available chromosomal domains between diploid and autotetraploid pak choi could help further develop a similar comprehension of other traits in other plants by combining the Hi-C method with RNA-seq technology in the future. In the post-genomic era, recognizing the layout of the chromosome in the nucleus, as well as its functional consequences, has emerged as an important priority (Kong and Zhang, 2019). In our work, we discovered that gene expression can be controlled through genetic changes and changes in spatial organization, as well as the identification of candidate genes that play a role in polyploid growth, which will help us breed *B. rapa* more effectively.

## Data Availability Statement

The original contributions presented in this study are publicly available. This data can be found here: PRJNA824220.

## Author Contributions

HW, CZ, XS, and XH conceived to the study. HW, YR, and CZ completed the experiments. HW, SL, and XS contributed to the data analysis and manuscript preparation. CZ, TL, YL, and XH participated in the planning of experiments and revising the manuscript. All authors read and approved the final version of the manuscript.

## Conflict of Interest

The authors declare that the research was conducted in the absence of any commercial or financial relationships that could be construed as a potential conflict of interest.

## Publisher’s Note

All claims expressed in this article are solely those of the authors and do not necessarily represent those of their affiliated organizations, or those of the publisher, the editors and the reviewers. Any product that may be evaluated in this article, or claim that may be made by its manufacturer, is not guaranteed or endorsed by the publisher.

## Footnotes

1
http://brassicadb.cn/#/
