# Supplementary material for: Integrated Analysis of Hi-C and RNA-Seq Reveals the Molecular Mechanism of Autopolyploid Growth Advantages in Pak Choi (Brassica rapa ssp. chinensis)
Source: Front Plant Sci. 2022 Jun 24;13:905202. doi: 10.3389/fpls.2022.905202 (PMC9263584; doi:10.3389/fpls.2022.905202)
Supplement: Supplementary file 1 [file Data_Sheet_1.docx]

**Supporting Figures**


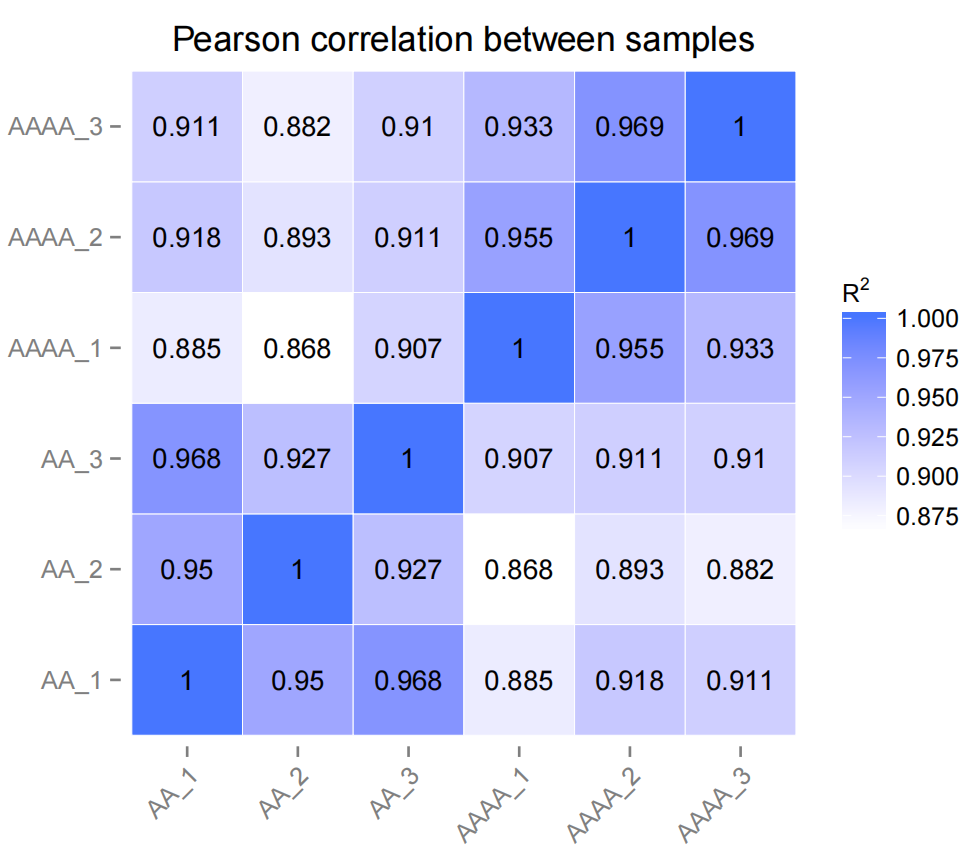


Figure S1. The correlation coefficient between each pair of diploid and autotetraploid pak choi biological replicates.

B

A


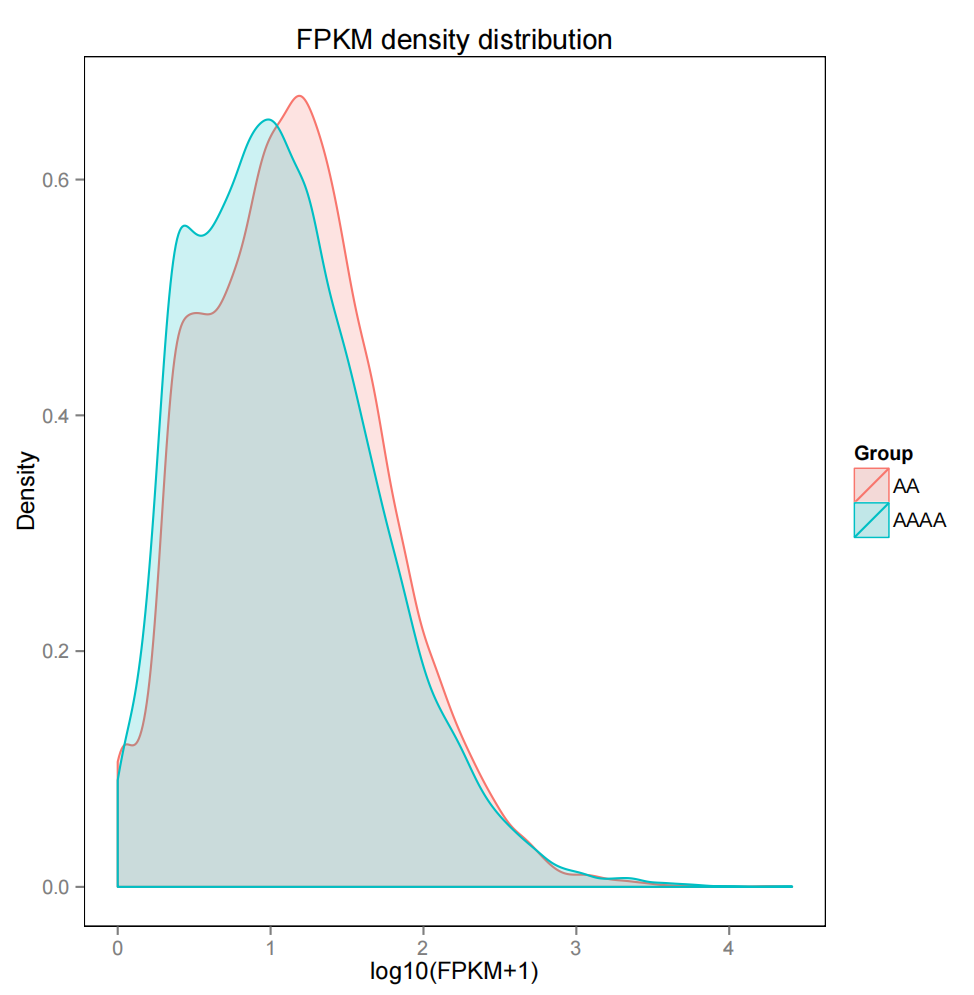

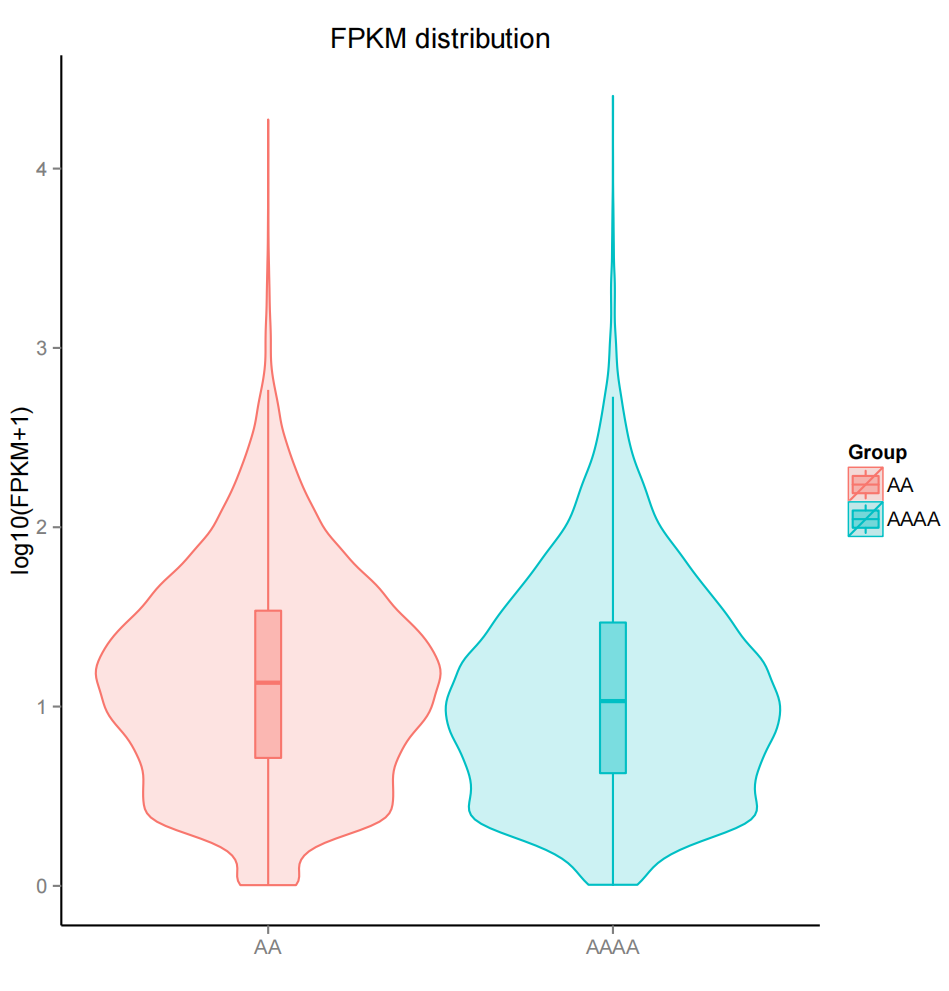


Figure S2. The distribution map (A) and violin diagram (B) of fragments per kilo-base of exon per million fragments mapped (FPKM).

1. Map of FPKM distribution. The horizontal coordinate is log_10_ (FPKM+1), and the vertical coordinate is the gene density. (B) Violin plot of FPKM. The horizontal coordinate is the sample name, the vertical coordinate is log_10_ (FPKM+1), and the width of each violin denotes the number of genes expressed at that level.


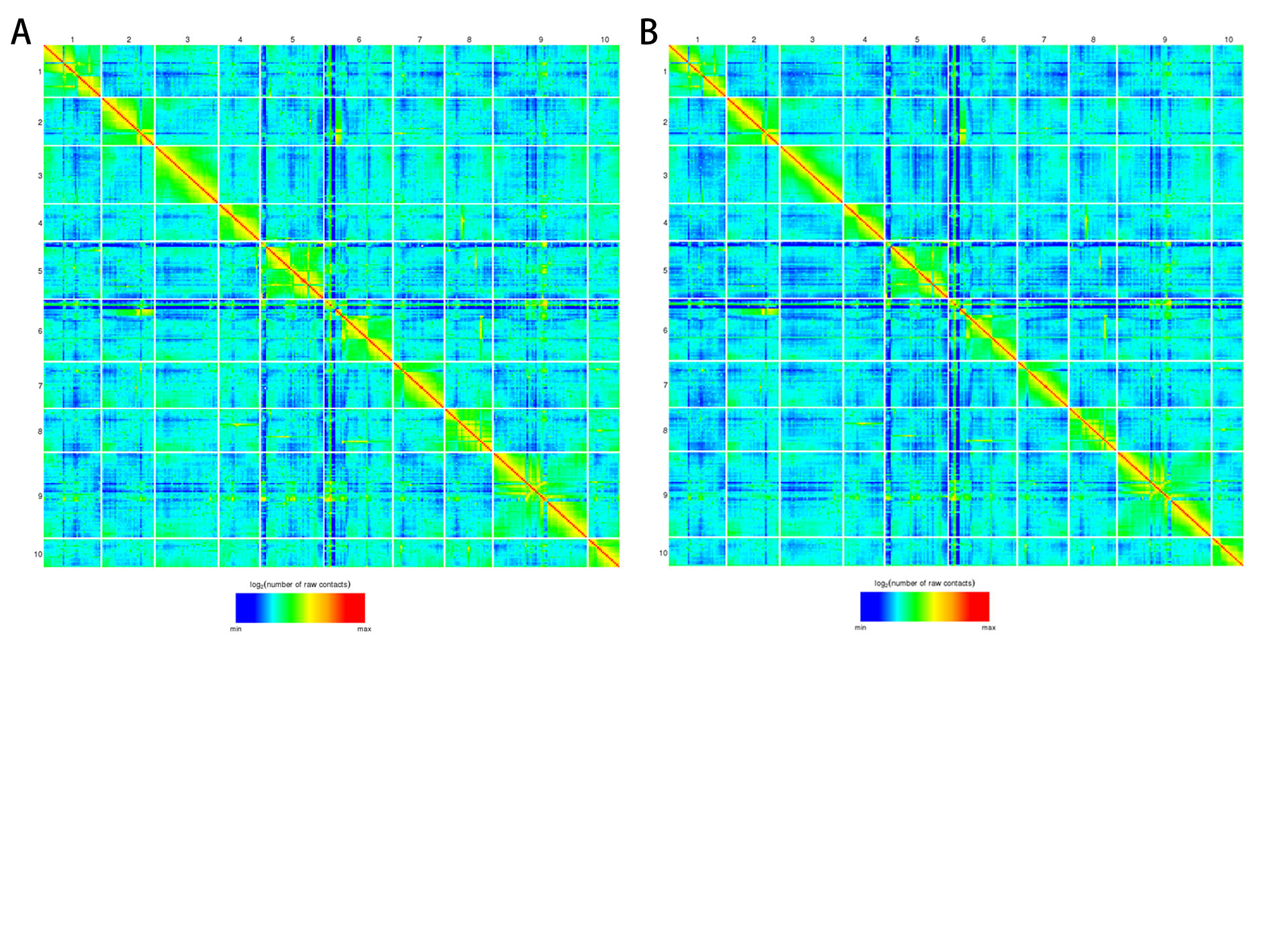


**Figure S3.** Genome-wide Hi-C contact maps for (A) diploid and (B) autotetraploid pak choi, at 1 Mb resolution. The matrix of heatmap represents the intra and interchromosomal interactions in the genome.

**
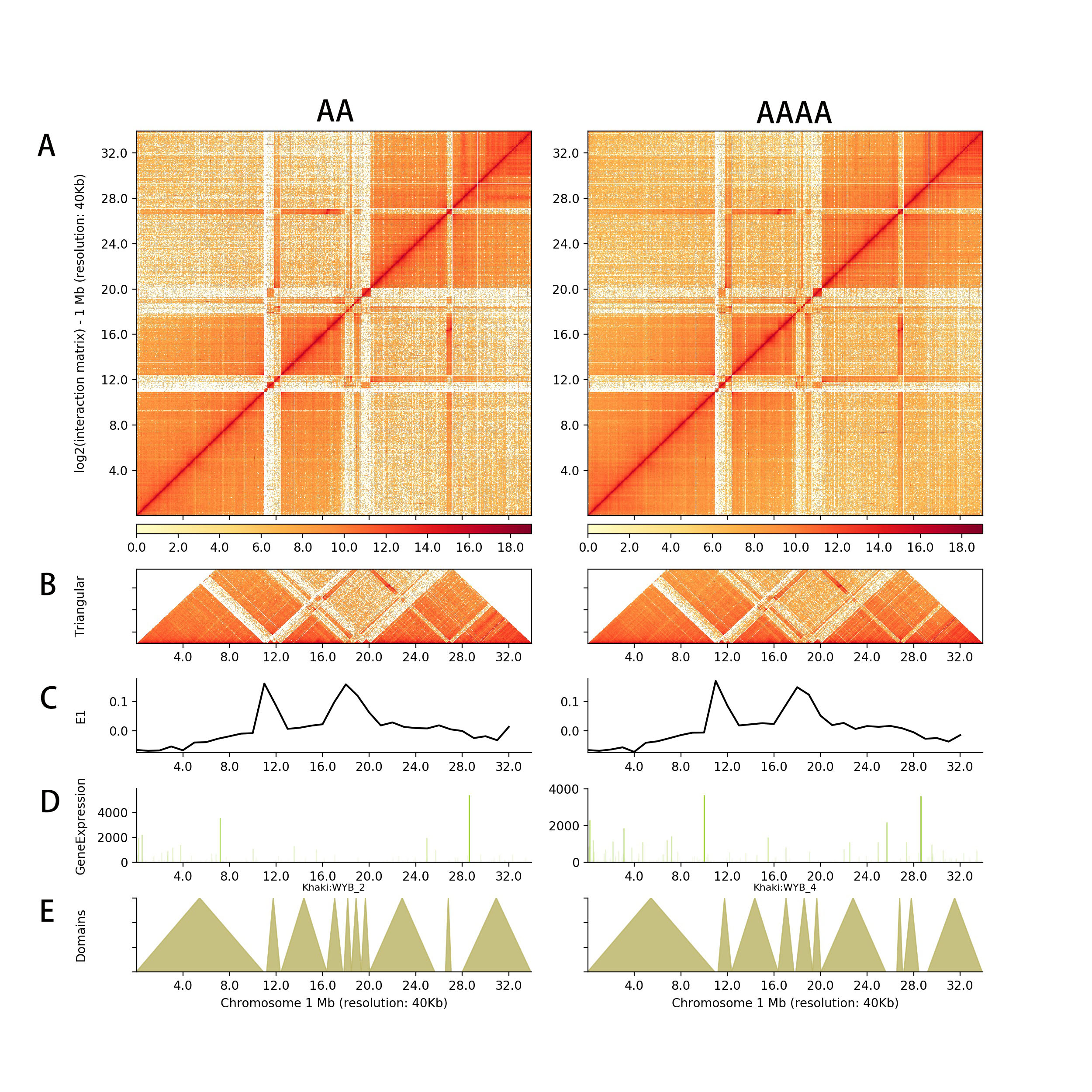

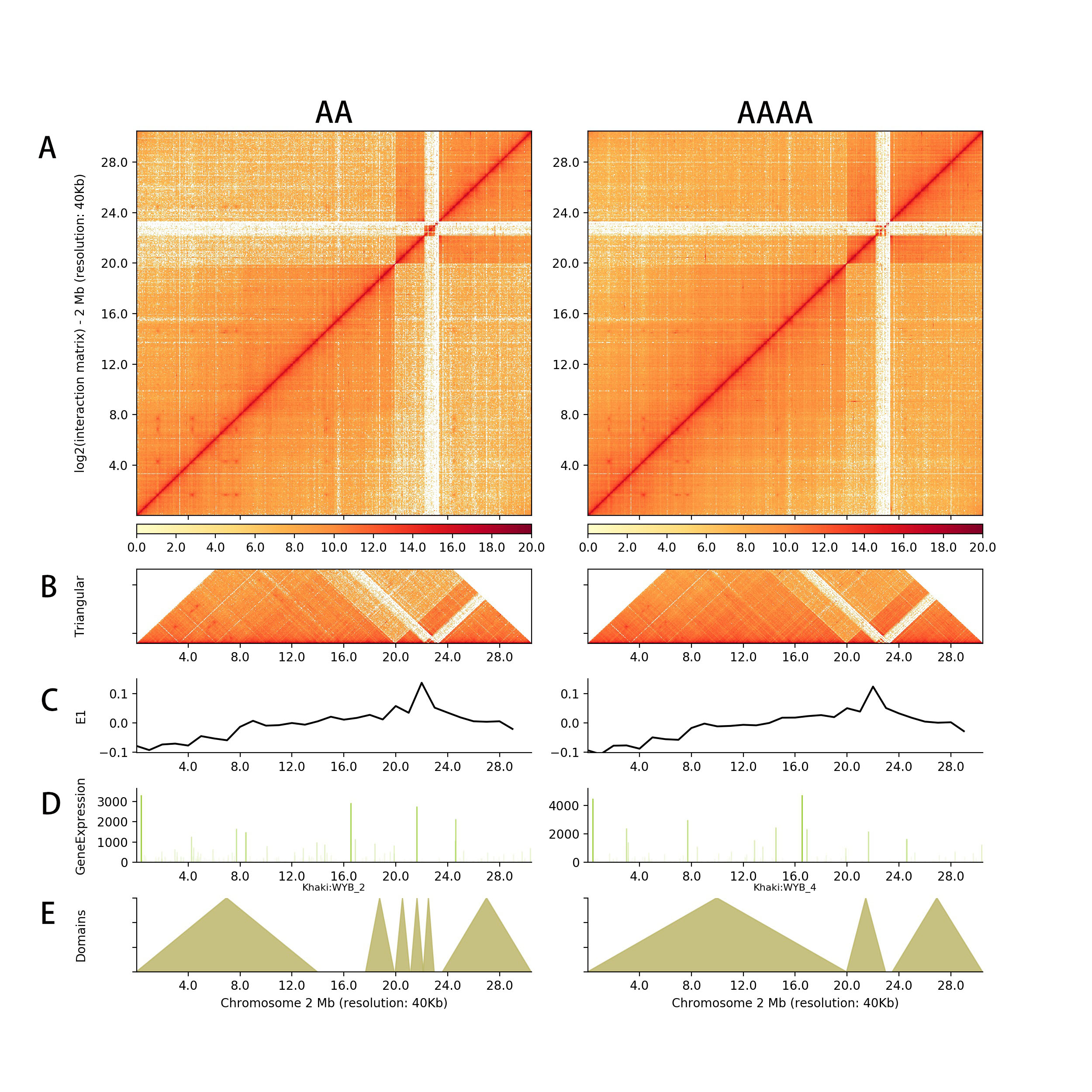

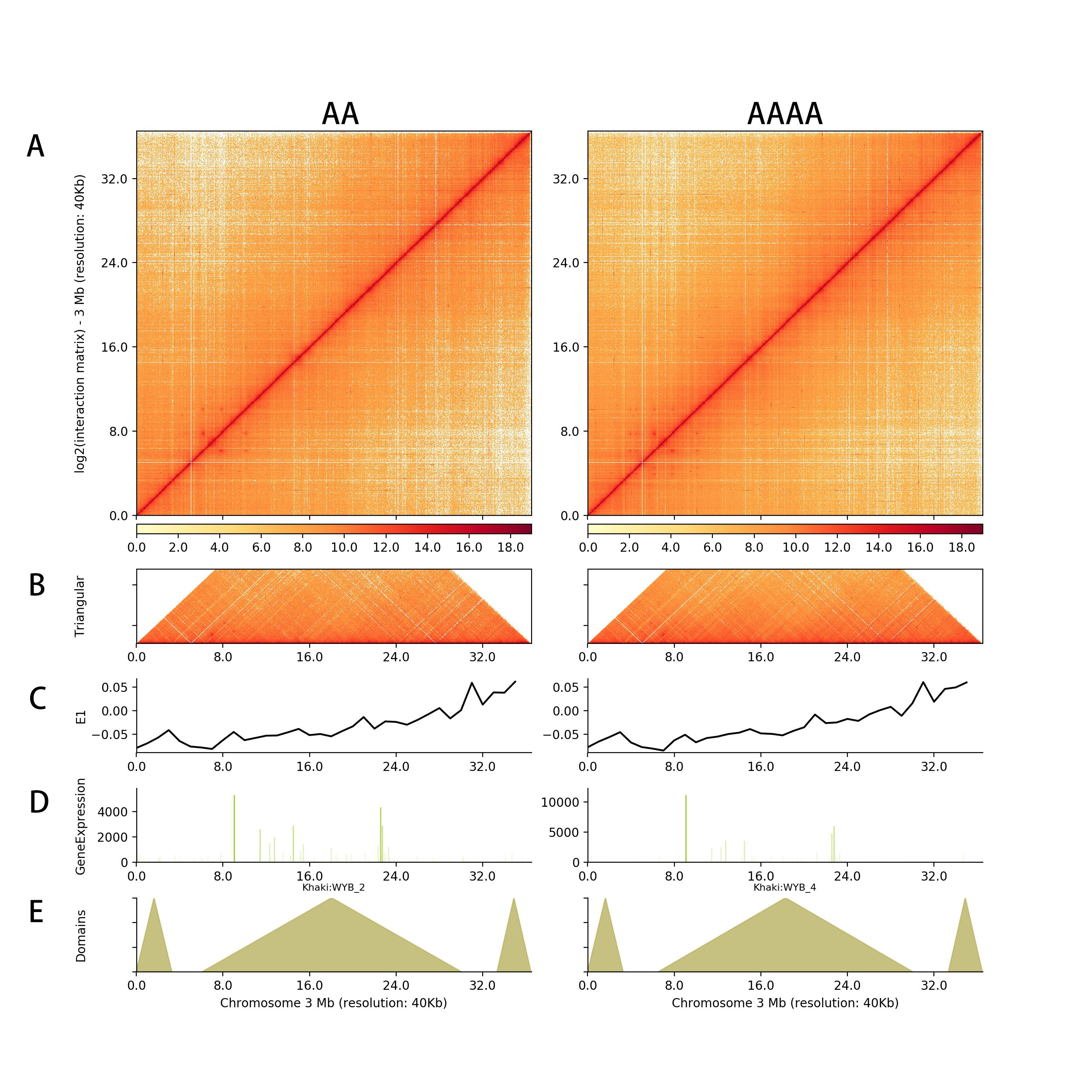

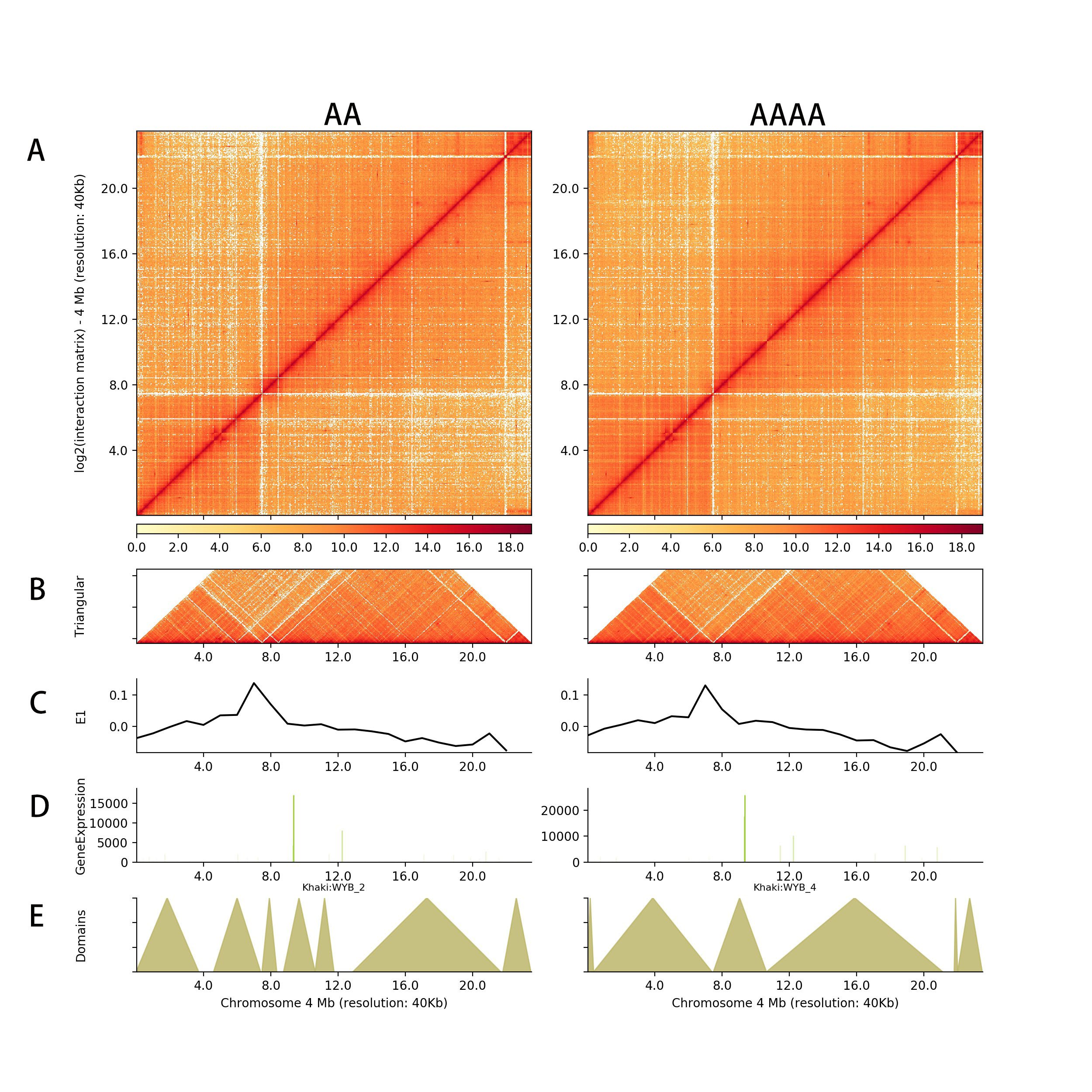

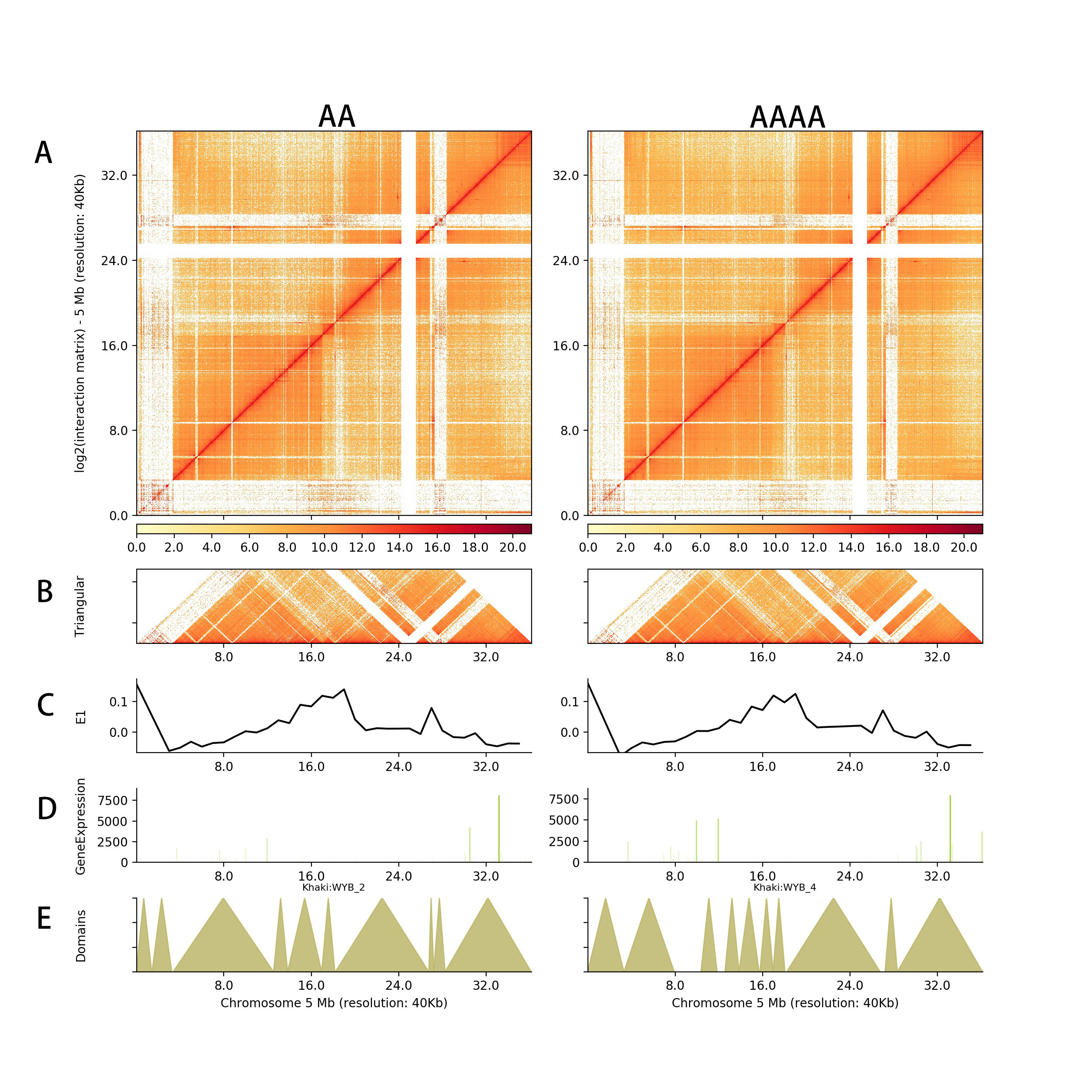

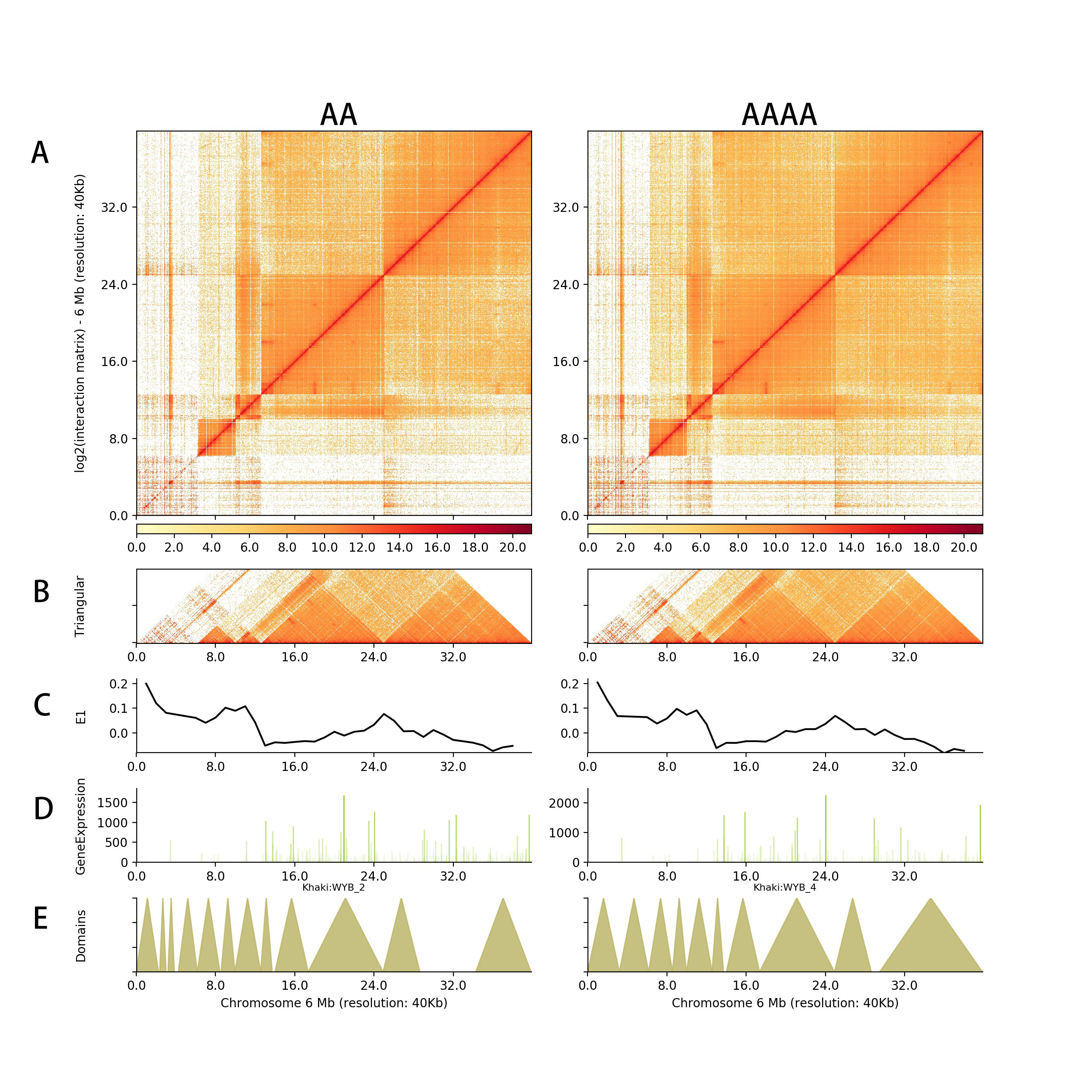

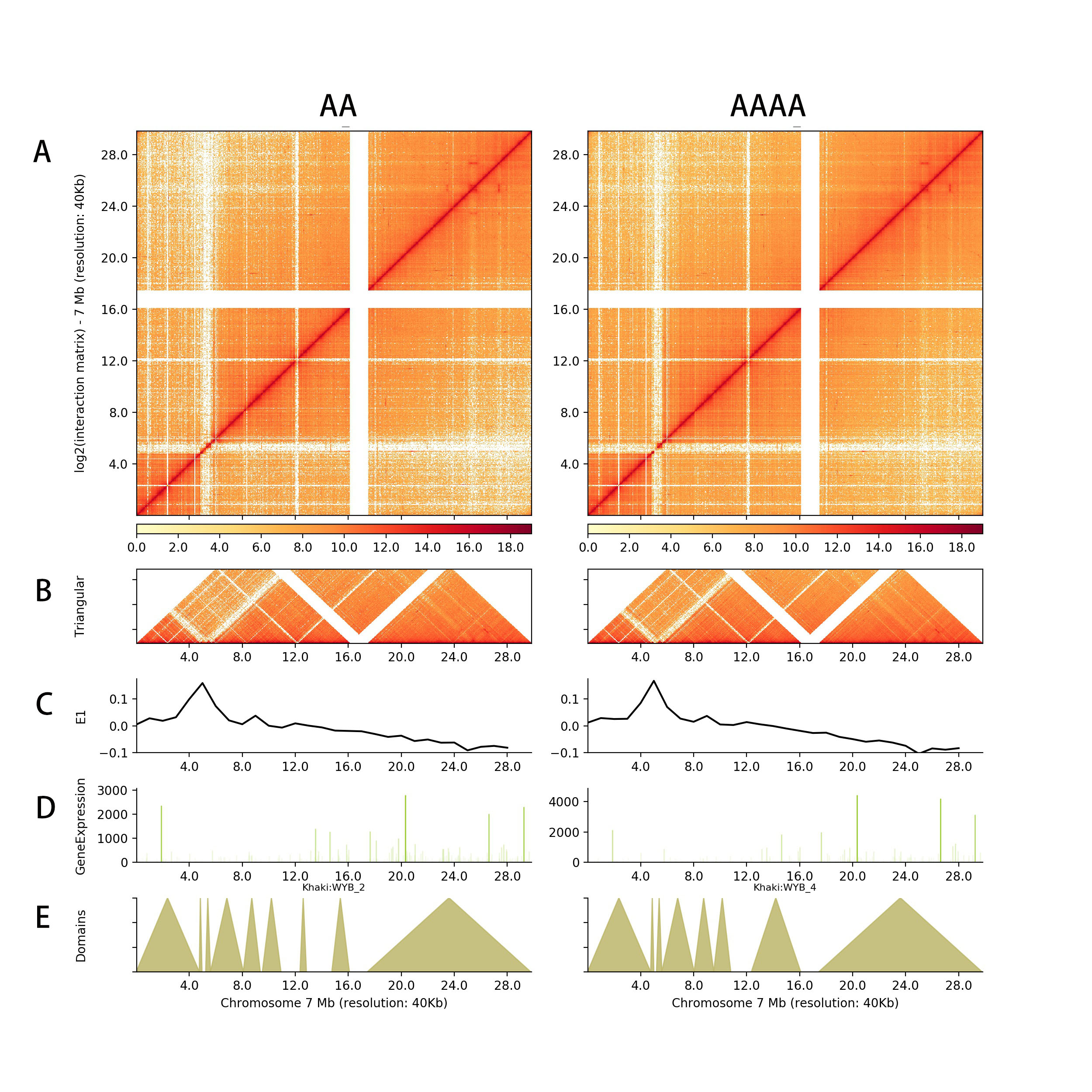

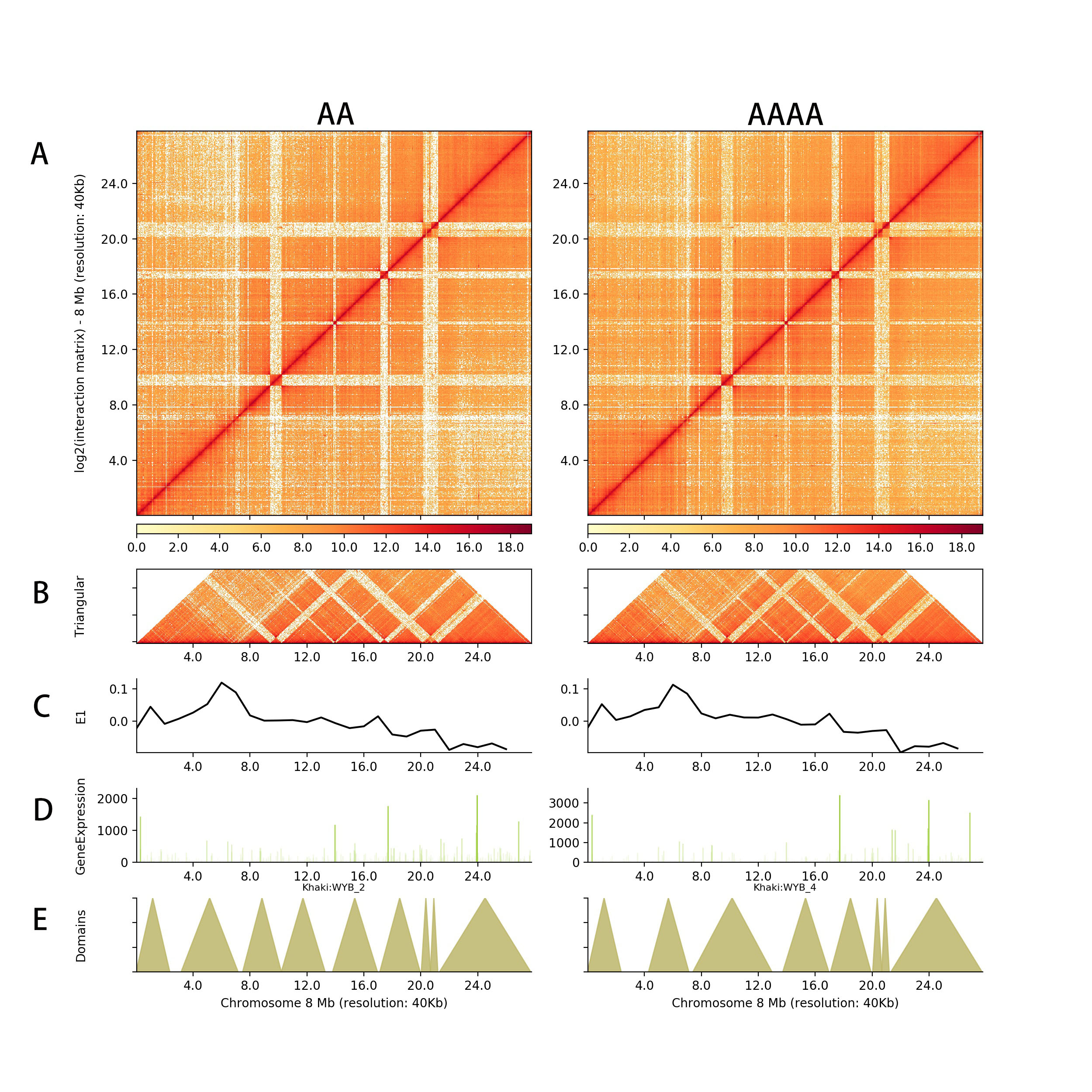

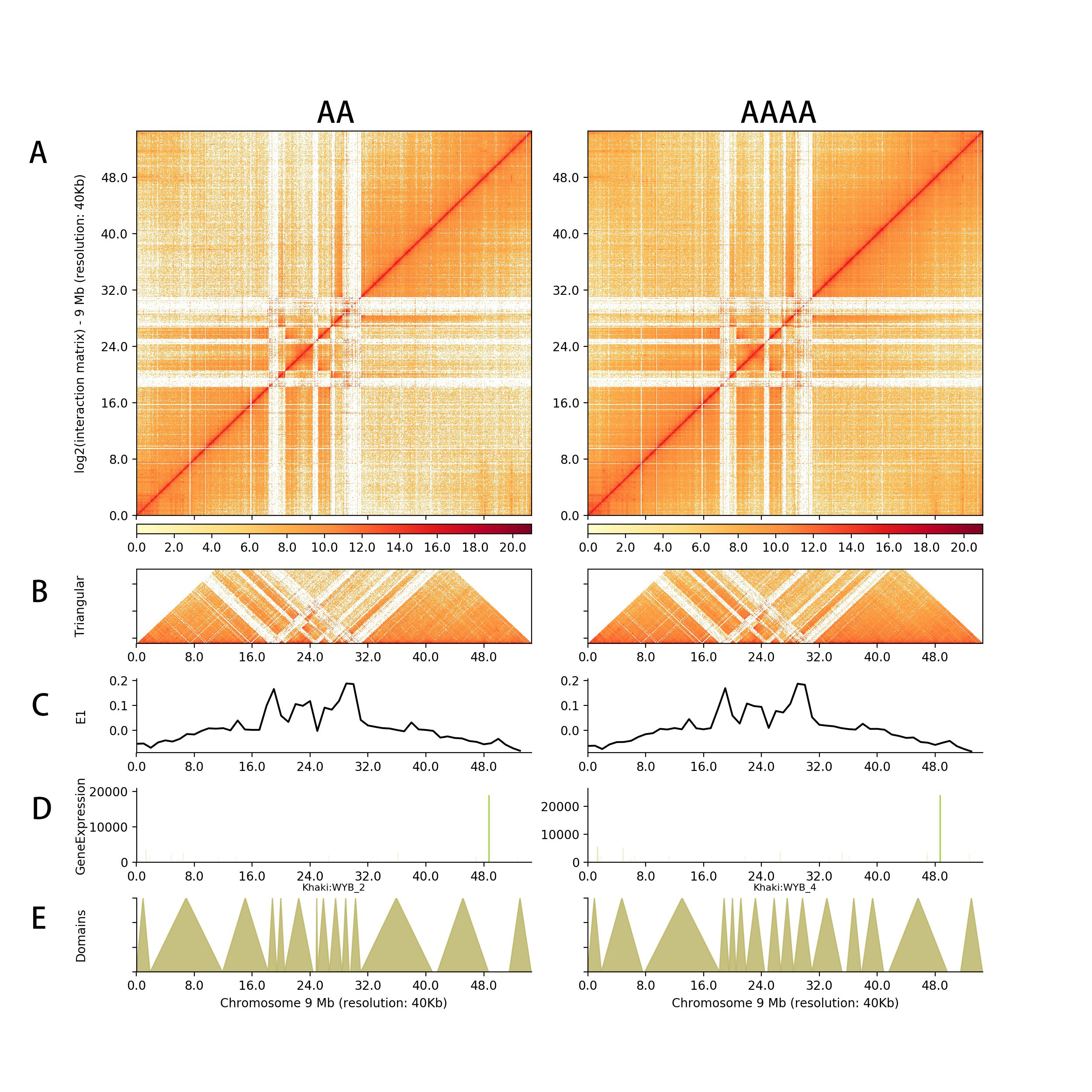

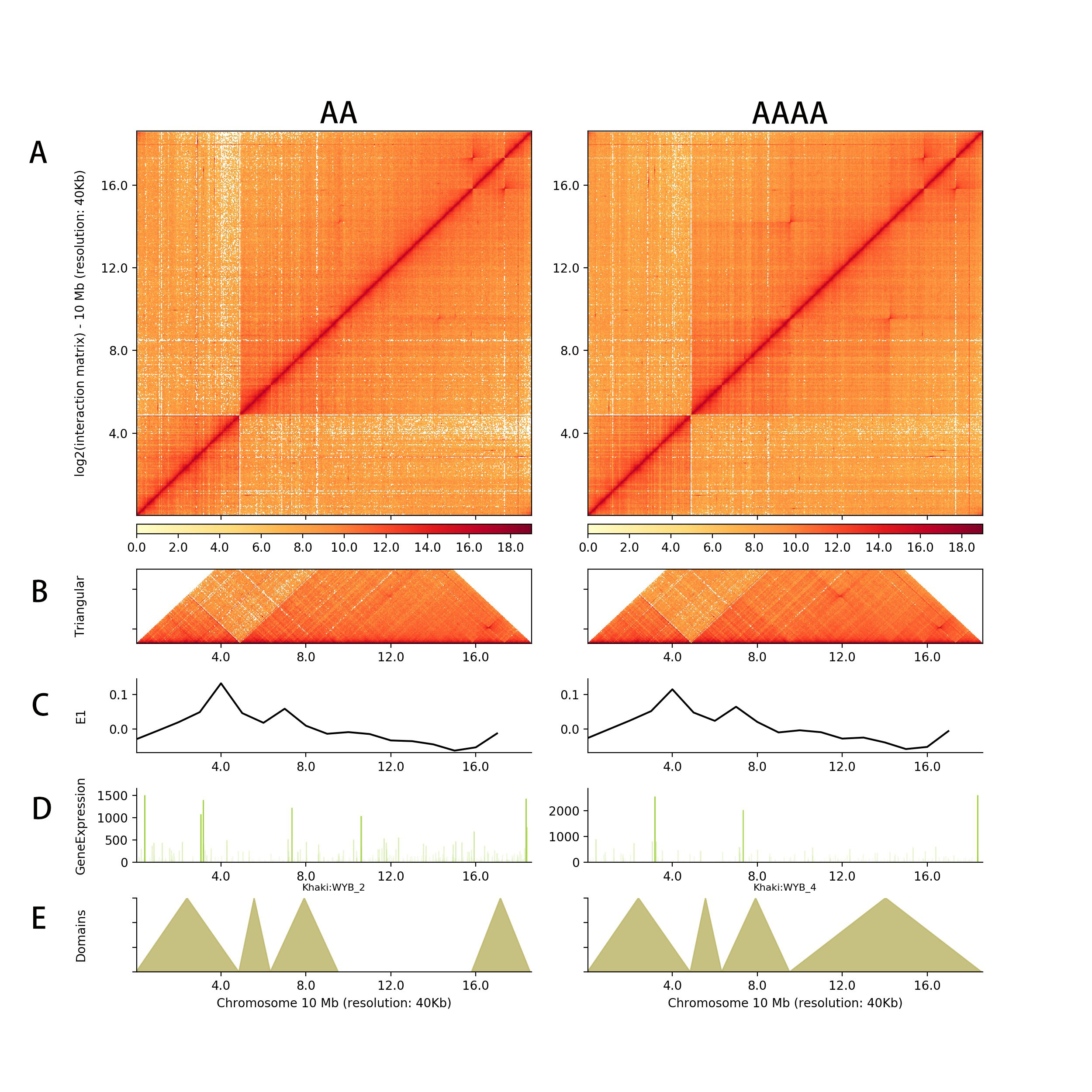
**

**Figure S4.** Analysis of 10 pairs of intra-chromosome interaction of diploid(AA) and autotetraploid(AAAA) samples.

1. Intrachromosomal interactions of chromosome at 40 Kb resolution. B. Each triangle distributed diagonally is represented as a topologically associated domain (TAD). C. First principal component values showing A/B compartment status. D. Gene expression level based on RNA-seq. E. Distribution of TADs.

**Figure S5.** Ratio of inter/intra interactions in diploid and autotetraploid pak choi.
